# Effect of pH-Responsive Charge-Conversional Polymer Coating to Cationic Reduced Graphene Oxide Nanostructures for Tumor Microenvironment-Targeted Drug Delivery Systems

**DOI:** 10.3390/nano9091289

**Published:** 2019-09-09

**Authors:** Kitae Ryu, Jaehong Park, Tae-il Kim

**Affiliations:** 1 Department of Biosystems & Biomaterials Science and Engineering, College of Agriculture and Life Sciences, Seoul National University, 1 Gwanak-ro, Gwanak-gu, Seoul 08826, Korea; 2 Research Institute of Agriculture and Life Sciences, Seoul National University, 1 Gwanak-ro, Gwanak-gu, Seoul 08826, Korea

**Keywords:** pH-responsive charge-conversion, polymer coating, graphene oxide, drug delivery systems, doxorubicin, serum stability

## Abstract

Tumor tissue represents a slightly acidic pH condition compared to normal tissue due to the accumulation of lactic acids via anaerobic metabolism. In this work, pH-responsive charge-conversional polymer (poly(ethylene imine)-poly(l-lysine)-poly(l-glutamic acid), PKE polymer) was employed for endowing charge-conversional property and serum stability to poly(ethylene imine) conjugated reduced graphene oxide-based drug delivery system (PEI-rGO). Zeta-potential value of PEI-rGO coated with PK_5_E_7_ polymer (PK_5_E_7_(PEI-rGO)) was −10.9 mV at pH 7.4 and converted to 29.2 mV at pH 6.0, showing pH-responsive charge-conversional property. Sharp-edged plate morphology of PEI-rGO was transformed to spherical nanostructures with vague edges by PK_5_E_7_ coating. Size of PK_5_E_7_(PEI-rGO) was found to be smaller than that of PEI-rGO in the serum condition, showing its increased serum stability. Loaded doxorubicin (DOX) in PK_5_E_7_(PEI-rGO) could be released rapidly in lysosomal condition (pH 5.0, 5 mM glutathione). Furthermore, DOX-loaded PK_5_E_7_(PEI-rGO) showed enhanced anticancer activity in HeLa and A549 cells in the tumor microenvironment-mimicking condition (pH 6.0, serum), which would be mediated by non-specific cellular interaction with decorated serum proteins. These results indicate that the pH-responsive charge-conversional PKE polymer coating strategy of cationic rGO nanostructures possesses a potential for acidic tumor microenvironment-targeted drug delivery systems.

## 1. Introduction

The tumor microenvironment does not simply refer to cancer cell clusters but also to the cancer-associated fibroblasts, myoblasts, epithelial cells, and immune cells along with the extracellular matrix (ECM), oxygen level, pH, and so on [1,2,3,4]. Cancer cells surrounded by the tumor microenvironment affect the cell growth, migration, neovascularization, and metastasis by secretion of growth factors as well as ECM degradation enzyme. Tumor tissue has a deficiency of oxygen and nutrition due to the rapid growth of cancer cells. Consequently, the tumor microenvironment turns to an acidic condition as the tissue metabolism progresses under anaerobic condition [5,6,7]. Therefore, accurate delivery of therapeutic drugs or genes to the target lesion based on the recognition of the acidic tumor microenvironment became an important research field for the development of gene or drug delivery systems and the treatment of cancer.

The main component that constitutes the serum protein is negatively-charged albumin [8]. Therefore, conventional cationic gene or drug delivery systems are prone to aggregate with negatively-charged serum protein due to the electrostatic interaction and to be eliminated via renal clearance or reticuloendothelial system [9]. In order to overcome this problem and utilize the acidic condition of the tumor microenvironment, the various strategies about gene or drug delivery systems using pH-responsive polymers have been pursued. In particular, maleic acid amide derivatives conjugated to amine groups which have pH-responsive charge-conversion ability [10,11,12,13,14,15,16,17], acid-labile linkages such as a hydrazone bond [18,19,20,21], and polypeptides which are composed of both cationic amino acids and anionic amino acids [22,23,24,25,26,27] have been utilized for gene or drug delivery systems. As a result, it is expected that these polymeric gene or drug delivery systems can enhance the delivery efficiency, shielding the interaction with negatively-charged serum proteins under normal physiological pH conditions and improving the cellular uptake by conversion to positively-charged systems through the specific pH-responsive charge-conversion behaviors in the acidic tumor environment (hydrolysis, protonation/deprotonation, etc.).

Graphene is a carbon-based material having a flat two-dimensional monolayer of the hexagonal honeycomb structure [28]. To obtain graphene nanosheets, the graphite oxide is prepared by Hummers’ method [29] and treated with ultrasonication to separate graphite oxide into a single layer of graphene oxide. The graphene oxide (GO) has various hydrophilic functional groups, such as hydroxyl groups (-OH), epoxide groups (-O-), and carboxyl groups (-COOH), which show high water dispersibility, chemical modifiability, and good biocompatibility. In addition, GO-based nanomaterials could be applied as hydrophobic drug delivery platforms due to their inherent characteristics in terms of drug loading via hydrophobic or π-π stacking interaction [30,31]. Especially, a GO derivative modified with branched poly(ethylene imine) (BPEI-GO) has been developed for gene delivery systems with low cytotoxicity and high transfection efficiency [32]. BPEI-GO also showed the potential as a bioimaging sensor due to its photoluminescence. In addition, poly(ethylene glycol)-conjugated PEI-modified reduced graphene oxide (PEG-BPEI-rGO) was synthesized for drug delivery systems [33]. It possessed much higher doxorubicin (DOX) loading efficiency than non-reduced derivative, PEG-BPEI-GO and showed high anticancer activity due to efficient endosome escape by endosome buffering and photothermal disruption of endosome membrane, and fast release of DOX by intracellular glutathione (GSH) and photothermal effect via near-infrared radiation.

Therefore, we assumed that this PEI-rGO-based nano drug delivery system would acquire the serum-resistance and the cancer cell killing ability, responding to acidic tumor microenvironment condition by coating with pH-responsive charge-conversional polymers.

Here, we used poly(ethylene imine)-poly(l-lysine)-poly(l-glutamic acid)s (PKE polymers) with various composition ratios of cationic amino acids and anionic amino acids as pH-responsive charge-conversional polymers [27] for the coating of PEI-rGO. PKE polymer nanostructures showed pH-responsive charge-conversion from negative charge at pH 7.4 to positive charges at pH 6.0, according to the protonation/deprotonation behavior of their lysine and glutamic acid residues. DOX was also employed as a drug molecule due to its feasible loading onto PEI-rGO nanostructures. DOX is a well-known anthracycline antibiotic with antineoplastic activity, which is isolated from the bacterium *Streptomyces peucetius caesius* [34].

In this study, we loaded DOX onto PEI-rGO and coated DOX-loaded PEI-rGO with PKE polymers, forming PKE(PEI-rGO/DOX) nanostructures. Then, physicochemical properties, serum stability, DOX release profile, and anticancer activity of PKE polymer-coated PEI-rGO/DOX nanostructures were examined based on pH-responsive charge-conversion ability.

## 2. Materials and Methods

### 2.1. Materials

Poly(ethylene imine) 1.8 kDa (branched, PEI_1.8k_) and l-glutathione (GSH) were purchased from Alfa Aesar (Haverhill, MA, USA). H-Lys(Z)-OH (Lys(Z)), l-Glutamic acid γ-benzyl ester (BGlu), trifluoroacetic acid, triphosgene, hydrobromic acid solution (33 wt% in acetic acid), agarose, and ethylenediaminetetraacetic acid (EDTA) were purchased from Sigma-Aldrich (St. Louis, MO, USA). Dimethylsulfoxide (DMSO), sodium chloride, and tris base were purchased Merck (Darmstadt, Germany). D_2_O and trifluoroacetic acid-D were purchased Cambridge Isotope Laboratories (Tewksbury, MA, USA). Tetrahydrofuran was purchased from Tokyo Chemical Industry (Tokyo, Japan). Doxorubicin (DOX) was purchased from Tocris Bioscience (Bristol, UK). Diethyl ether and n-hexane were purchased from Daejung (Siheung, Korea). Thiazolyl blue tetrazolium bromide was purchased from Gold Bio (St. Louis, MO, USA). Dulbecco’s Modified Eagles’ Medium (1X) + GlutaMAX-1 (DMEM), Dulbecco’s Phosphate Buffered Saline (DPBS), 0.25% Trypsin-EDTA, fetal bovine serum (FBS), and Penicillin-Streptomycin (Pen Strep, P/S) were purchased from Invitrogen-Gibco (Carlsbad, CA, USA). All other chemicals were purchased and used without any further purification.

### 2.2. Synthesis of PKE Polymers

PKE polymers were synthesized using PEI_1.8k_ as a macroinitiator by ring-opening polymerization of amino acid N-carboxyanhydride (NCA) as previously described [27]. Briefly, Lys(Z)-NCA and BGlu-NCA monomer were prepared using triphosgene ring formation reaction. Lys(Z)-NCA (50 molar excess to PEI) was reacted with PEI_1.8k_ to synthesize PEI_1.8k_-polyLys(Z) and subsequently, BGlu-NCA (50, 70, 90, or 130 molar excess to PEI) was reacted with PEI_1.8k_-polyLys(Z) to synthesize PEI_1.8k_-polyLys(Z)-polyBGlu. After removal of protecting groups, poly(ethylene imine)-poly(l-lysine)-poly(l-glutamic acid)s (PKE polymers) were obtained. The synthesized PKE polymers were named as PK_5_E_5_, PK_5_E_7_, PK_5_E_9_, and PK_5_E_13_, respectively, based on the ratios of conjugated l-lysine and l-glutamic acid moieties to PEI_1.8k_.

### 2.3. Preparation of DOX-Loaded PEI-rGO (PEI-rGO/DOX)

PEI-conjugated reduced graphene oxide (PEI-rGO) [33] was kindly provided by Prof. Won Jong Kim in Pohang University of Science and Technology, Korea. DOX was dissolved in DMSO at a concentration of 5 mg/mL. The DOX/DMSO solution (50 wt% to PEI-rGO) was slowly added to the PEI-rGO solution. The mixture solution was shaken for 12 h in dark condition. Then, dialysis (MWCO: 3000) was performed for 24 h to remove unbound DOX. Finally, PEI-rGO/DOX was obtained through centrifugation (4000 rpm, 20 min, 25 °C) using Amicon Ultra-4 centrifugal filters 3K (Merck, Germany).

The DOX loading in PEI-rGO was confirmed by measuring absorbance (300–700 nm) of PEI-rGO and PEI-rGO/DOX respectively, using a microplate reader (Synergy H1, BioTek, Winooski, VT, USA). The DOX loading efficiency was examined by measuring DOX fluorescence (Ex: 480 nm, Em: 520–700 nm) of PEI-rGO/DOX in water or 90% DMSO. Ten microliters (10 μL) of PEI-rGO/DOX solution (1 mg/mL PEI-rGO) was added to 90 μL of water or DMSO. The DOX loading amount was calculated by comparison of DOX fluorescence values measured in water and 90% DMSO. DOX loading efficiency was calculated as below:

(1) $$\begin{array}{c} \mathrm{Drug}\;\mathrm{loading}\;\mathrm{efficiency}\;(\mathrm{DLE},\;\%)\\ \;=(\frac{\mathrm{Weight}\;\mathrm{of}\;\mathrm{loaded}\;\mathrm{DOX}\;(\mathrm{mg})}{\mathrm{Weight}\;\mathrm{of}\;\mathrm{total}\;\mathrm{DOX}\;\mathrm{for}\;\mathrm{loading}\;(\mathrm{mg})})\times 100 \end{array}$$

### 2.4. Preparation of PKE Polymer-Coated PEI-rGO (PKE(PEI-rGO)) and PKE Polymer-Coated PEI-rGO/DOX (PKE(PEI-rGO/DOX))

To form PKE(PEI-rGO) or PKE(PEI-rGO/DOX) nanostructures, PEI-rGO or PEI-rGO/DOX solution (deionized water) were added to PKE polymer solutions (deionized water) having pre-determined weight ratios (PKE:PEI-rGO = 10:1, 20:1, 30:1, or 50:1, *w*/*w*) by dropwise, respectively. The mixtures were incubated for 1 h with orbital shaking at room temperature. Then, they were centrifuged (4000 rpm, 10 min, room temperature) and re-dispersed in solutions after removal of supernatants.

### 2.5. Characterization of PEI-rGO Based Nanostructures

The average sizes and zeta-potential values of the prepared PKE(PEI-rGO) and PKE(PEI-rGO/DOX) nanostructures were measured by using a Zeta-sizer Nano ZS90 (Malvern Instruments, Malvern, UK). First, the average sizes and zeta-potential values of PKE polymer-coated PEI-rGOs (PKE(PEI-rGO)) with various PKE polymer weight ratios (10, 20, 30, or 50) were measured. Additionally, the average size and zeta-potential value of each PKE(PEI-rGO) nanostructure (weight ratio = 30) were measured at pH 7.4 and pH 6.0. Additionally, the average sizes and zeta-potential values of PK_5_E_7_(PEI-rGO/DOX) and PK_5_E_9_(PEI-rGO/DOX) (weight ratio = 30) were measured at pH 7.4 and pH 6.0 to examine the property change after DOX loading to the PEI-rGO.

The energy filtered transmission electron microscope (EF-TEM) was used to observe the morphology of PEI-rGO and PEI-rGO/DOX nanostructures with and without PK_5_E_7_ polymer coating (weight ratio = 30). The samples were absorbed onto TEM copper grid. After drying for 12 h and the images were visualized by EF-TEM (LIBRA 120, Carl Zeiss, Oberkochen, Germany) under the voltage of 80 kV.

### 2.6. Serum Stability of PKE Polymer Coated PEI-rGO (PKE(PEI-rGO))

The nanostructure stability in serum condition was investigated by measuring the size changes of PEI-rGO based nanostructures. PEI-rGO or PK_5_E_7_(PEI-rGO) was centrifuged (4000 rpm, 10 min, room temperature) and dispersed in PBS or PBS containing 10% FBS at pH 7.4 or 6.0. Each sample was incubated at 37 °C with gentle shaking. After pre-determined incubation time (2, 4, 8, 16, and 24 h), the average size of each sample was measured using Zeta-sizer.

### 2.7. DOX Release Profile of PK_5_E_7_(PEI-rGO/DOX)

DOX release profile of PK_5_E_7_ coated PEI-rGO/DOX was examined. One-hundred micrograms (100 μg) of PK_5_E_7_(PEI-rGO/DOX) (weight ratio = 30) was prepared for measuring DOX release. The total volume of PK_5_E_7_(PEI-rGO/DOX) solution was adjusted to 2 mL and the solution was placed in a dialysis membrane (MWCO: 3000) in 50 mL of PBS (pH 7.4, pH 5.0, or pH 5.0 with 5 mM of glutathione). The solutions were incubated at 37 °C with gentle shaking in dark condition. After pre-determined incubation time (0.5, 1, 2, 4, 8, 12, and 24 h), released DOX amount was analyzed by measuring the DOX fluorescence outside the membrane (Ex = 480 nm, Em = 560 nm) using a microplate reader.

### 2.8. Anticancer Activity of PK_5_E_7_(PEI-rGO/DOX)

Anticancer activity of PK_5_E_7_(PEI-rGO/DOX) was examined by MTT assay in human cervical adenocarcinoma cells (HeLa cells) and human lung adenocarcinoma epithelial cells (A549 cells). HeLa and A549 cells were cultured in DMEM with 10% FBS and 1% penicillin/streptomycin (P/S) (37 °C, 5% CO_2_). Cells were seeded at 1 × 10^4^ cells/well on 96-well cell culture plates and cultured for 24 h. After reaching 70–80% of cell confluency, the pH of the medium was adjusted to pH 7.4 or 6.0. One-hundred microliters (100 μL) of PK_5_E_7_(PEI-rGO/DOX) solution (10, 20, or 50 μg/mL DOX) was treated to each well and incubated for 24 h. DOX only, PK_5_E_7_, and PK_5_E_7_(PEI-rGO) with various concentration (pH 7.4) were also treated to HeLa cells or A549 cells as controls. Then, each medium was replaced with fresh medium containing 10% FBS and 1% P/S. After further incubation for 24 h, 25 μL of MTT/DPBS solution (2 mg/mL) was added to each well and incubated for 2 h. The medium was carefully removed and 150 μL of DMSO was added to the formazan crystal formed by proliferating cells. The absorbance at 570 nm was measured using a microplate reader. The anticancer activity was expressed as the relative cell viability (%) by comparing the absorbance values with cell only value.

### 2.9. Cellular Uptake of PKE Polymer Coated PEI-rGO/DOX 

The cellular uptake efficiency of PK_5_E_7_(PEI-rGO/DOX) (weight ratio = 30) was evaluated by flow cytometry analysis in HeLa and A549 cells. Cells were seeded at a density of 2 × 10^5^ cells/well on six-well cell culture plates. Having achieved 70–80% of confluency, the cells were treated with PK_5_E_7_(PEI-rGO/DOX) solutions in serum-free or serum-containing medium (pH 7.4 or 6.0) for 4 h, respectively. Then, the cells were washed twice with ice-cold DPBS and detached using trypsin-EDTA. The cells were resuspended in DPBS and analyzed by FACS using BD Accuri C6 flow cytometer (Becton Dickinson Biosciences, San Jose, CA, USA) at a minimum of 1 × 10^4^ cells gated per sample. The flow cytometry results were processed by Flowing software (Cell Imaging Core, Turku Centre for Biotechnology, Turku, Finland).

### 2.10. Fluorescence Microscopy Observation of PK_5_E_7_(PEI-rGO/DOX)

A549 cells were seeded at a density of 2 × 10^5^ cells/well on six-well cell culture plates. Having achieved 70–80% of confluency, the cells were treated with PK_5_E_7_(PEI-rGO/DOX) solutions (10 μg/mL DOX) in serum-free or serum-containing medium (pH 7.4 or 6.0) for 24 h, respectively. Then, the cells were washed twice with ice-cold DPBS and stained with Hoechst 33342 (10 μg/mL) for 15 min. After washing with DPBS twice, fluorescence images were observed by fluorescence microscope (iRiS Digital Cell Imaging System, Logos Biosystems, Anyang, Korea).

## 3. Results and Discussions

### 3.1. Formation of PKE Polymer Coated PEI-rGO (PKE(PEI-rGO))

According to the previous study [27], it was confirmed that 51.8 l-lysine residues were conjugated to one PEI1.8k molecule and conjugated l-glutamic acid residues in PKE polymers were 53.4, 75.9, 93.7, and 140.5 molecules per one PEI1.8k molecule, respectively. According to the ratios of conjugated l-lysine and l-glutamic acid moieties to PEI1.8k, PKE polymers were named as PK_5_E_5_, PK_5_E_7_, PK_5_E_9_, and PK_5_E_13_, respectively. PKE polymers showed pH-responsive charge-conversional ability, according to the protonation/deprotonation behavior of their lysine and glutamic acid residues responding to environmental pH change. For example, the zeta-potential value of PK_5_E_9_ polymer particle was increased from −35.0 mV to 27.5 mV as pH was decreased from 10.0 to 3.0. This change could induce the increase of both cellular uptake and serum stability in terms of gene delivery systems. The inherent characteristics of the pH-responsive charge-conversional ability of PKE polymers could be regulated via controlling the ratios between l-lysine and l-glutamic acid residues.

Therefore, it was assumed that we would modulate the physicochemical properties of PEI-rGO by coating with PKE polymers via electrostatic interaction for improvement of serum stability and anticancer efficacy responding to environmental pH.

First, Z-average sizes and zeta-potential values of PKE(PEI-rGO) nanostructures with various weight ratios between PKE and PEI-rGO were measured to examine the PKE coating effect to PEI-rGO. It was found that intact PEI-rGO without coating had about 200 nm size and 50 mV zeta-potential value.

**Figure 1:**
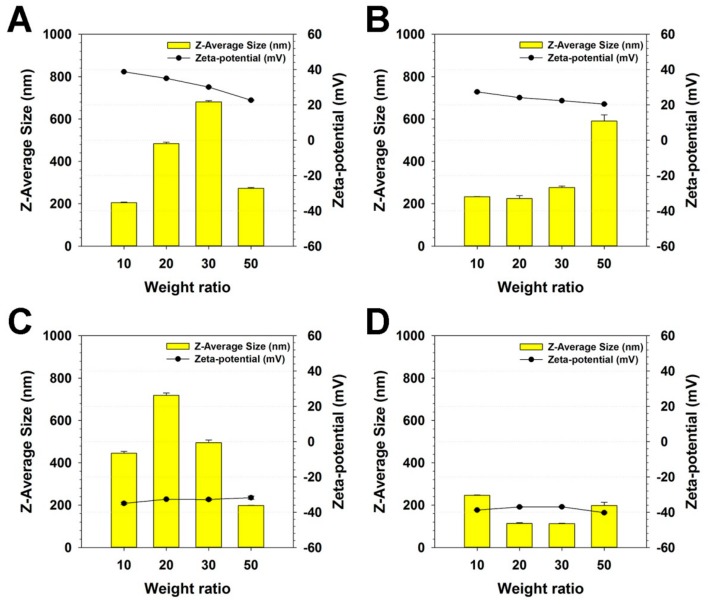
Z-Average sizes and zeta-potential values of (**A**) PK_5_E_5_(PEI-rGO), (**B**) PK_5_E_7_(PEI-rGO), (**C**) PK_5_E_9_(PEI-rGO), and (**D**) PK_5_E_13_(PEI-rGO) with various weight ratios (PKE:PEI-rGO). Data are shown as the mean ± S.D (*n* = 3).

As shown in Figure 1A, the sizes of PK_5_E_5_(PEI-rGO) were gradually increased from 204.7 nm to 681.3 nm with the increase of weight ratios from 10 to 30 and then decreased to 272.5 nm at a weight ratio of 50. Zeta-potential values of PK_5_E_5_(PEI-rGO) were gradually decreased from 38.7 mV to 22.7 mV with the increase of the weight ratios from 10 to 50. In the case of PK_5_E_7_ coating to PEI-rGO (Figure 1B), the sizes were maintained under 300 nm at weight ratios of 10–30 (225.1~277.1 nm) and the size was greatly increased at a weight ratio of 50 (590.6 nm). In Figure S1, it was found that PK_5_E_7_(PEI-rGO) showed homogeneous size distributions, regardless of weight ratios. Their zeta-potential values were reduced from 27.4 to 20.5 mV with the increase of weight ratios from 10 to 50. The size of PK_5_E_9_(PEI-rGO) was the largest (718.5 nm) at a weight ratio of 20 (Figure 1C). At weight ratios of 30 and 50, the sizes were gradually decreased to 495.0 nm and 198.0 nm, respectively. PK_5_E_9_(PEI-rGO) showed zeta potential value of −35.0 at a weight ratio of 10, which remained unchanged significantly (−32.8~−31.8 mV) regardless of weight ratios. PK_5_E_13_(PEI-rGO) displayed sizes less than 250 nm (113.03~246.6 nm) and zeta-potential values maintaining negative values (−40.3~−37.0 mV), regardless of weight ratios (Figure 1D).

These results suggested that PKE(PEI-rGO) nanostructures could form aggregates at certain coating weight ratios of PKE polymers, which were dependent on PKE polymer types. It is thought that PKE polymers with different cationic lysine and anionic glutamic acid ratios, which means the different net charges and chain lengths, would act as electrostatic glues between the nanostructures for aggregate formation during coating process [35].

Decreased zeta-potential values of PK_5_E_5_(PEI-rGO) and PK_5_E_7_(PEI-rGO) with the increase of PKE weight ratios mean that anionic glutamic acid residues of PKE polymers would induce the negative surface charges to PEI-rGO by coating. In the case of PK_5_E_9_(PEI-rGO) and PK_5_E_13_(PEI-rGO), their negative zeta-potential values mean that negative charges from glutamic acid residues exceed positive charges from lysine residues due to their having more anionic moieties (carboxylic acid of glutamic acid) than cationic moieties (amine of PEI and lysine).

### 3.2. pH-Responsive Charge-Conversional Ability of PKE(PEI-rGO)s

It was expected that PKE(PEI-rGO) nanostructures would possess pH-responsive charge-conversional property, which means the maintenance of negatively charged surfaces at normal physiological pH, conferring serum stability and conversion to a positively charged surfaces in mild acidic condition like tumor microenvironment, providing improved adsorption to negatively-charged cellular membrane and cellular uptake. Therefore, Z-average sizes and zeta-potential values of PKE(PEI-rGO) nanostructures were examined at pH 7.0 and 6.4.

**Figure 2:**
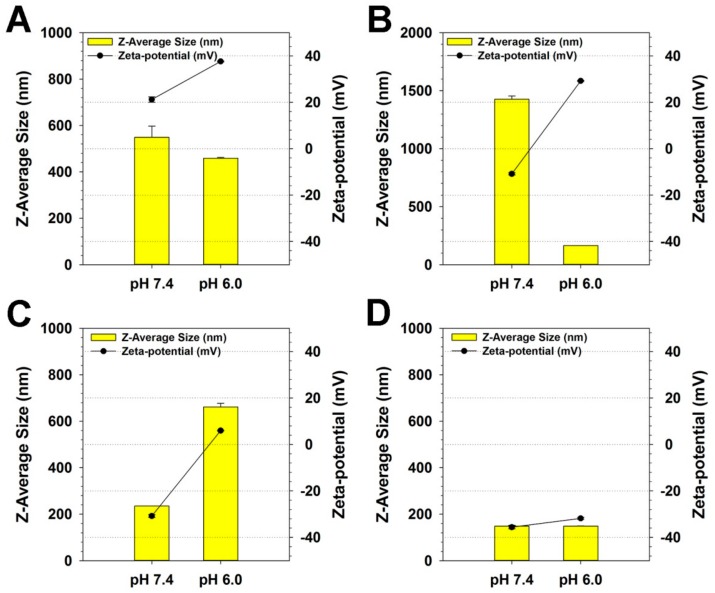
Z-average sizes and zeta-potential values of PKE(PEI-rGO) nanostructures at pH 7.4 and 6.0. (**A**) PK_5_E_5_(PEI-rGO), (**B**) PK_5_E_7_(PEI-rGO), (**C**) PK_5_E_9_(PEI-rGO), and (**D**) PK_5_E_13_(PEI-rGO). The PKE polymer coating ratio was set to a weight ratio of 30. Data are shown as the mean ± S.D (*n* = 3).

As shown in Figure 2A, the size of PK_5_E_5_(PEI-rGO) at pH 7.4 (548.7 nm) was slightly decreased to 459.0 nm at pH 6.0, while its zeta-potential at pH 7.4 (21.2 mV) was increased to 37.6 mV at pH 6.0. PK_5_E_7_(PEI-rGO) presented 1425.7 nm size probably due to the aggregates formation during coating process and −10.9 mV zeta-potential value at pH 7.4 (Figure 2B). However, its size was dramatically decreased to 164.3 nm and the zeta-potential was converted to positive value (29.2 mV) at pH 6.0. In Figure 2C, PK_5_E_9_(PEI-rGO) exhibited 235.2 nm size and −30.8 mV zeta-potential value at pH 7.4. At pH 6.0, its size was increased to 661.6 nm and the zeta-potential value was 6.0 mV. In the case of PK_5_E_13_(PEI-rGO), the size (about 150 nm) and the zeta-potential value (−35.6~−31.8 mV) were not significantly changed, regardless of pH.

These results indicated that only PK_5_E_7_(PEI-rGO) and PK_5_E_9_(PEI-rGO) nanostructures possessed pH-responsive charge-conversional properties, due to the charge balance between carboxylic acid groups of glutamic acid residues and amines of lysine residues and PEI. In these pH conditions (pH 7.4 and 6.0), ε-amines of lysine and primary amines of PEI (pKa = ~10) would be almost protonated, contributing to the positive zeta-potential values of PKE(PEI-rGO) nanostructures, while carboxylic acid groups of glutamic acid residues and other amines of PEI (secondary and tertiary) would modulate net zeta-potential values, displaying pH-dependent protonation/deprotonation behaviors. It was found that coating by PK_5_E_5_ polymer with the least glutamic acid portion and PK_5_E_13_ polymer with the most glutamic acid portion constructed positively-charged and negatively-charged nanostructures, respectively, at both pH 7.4 and pH 6.0, which means that they lack pH-responsive charge-conversional properties. Although the PK_5_E_9_(PEI-rGO) also exhibited the charge-conversional ability, it was presumed that the PK_5_E_9_(PEI-rGO) nanostructure would be difficult to access to the cell membrane due to the large particle size and the low surface charge value at pH 6.0.

### 3.3. Characterization of PKE(PEI-rGO/DOX)s

**Figure 3:**
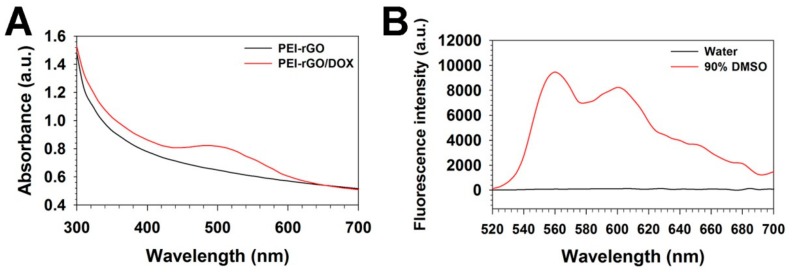
(**A**) Absorbance spectra of PEI-rGO (black) and PEI-rGO/DOX (red). (**B**) Fluorescence spectra of PEI-rGO/DOX in water (black) and 90% DMSO (red).

First, the drug loading efficiency of PEI-rGO was assessed by measurement of absorbance and fluorescence of DOX-loaded PEI-rGO (PEI-rGO/DOX). As shown in Figure 3A, the characteristic absorbance peak of DOX was observed at 500 nm in PEI-rGO/DOX in contrast to PEI-rGO displaying no DOX peak, which means DOX molecules were successfully loaded on the surface of PEI-rGO by hydrophobic and π-π stacking interaction between DOX and rGO as previously reported [33]. In fluorescence analysis (Figure 3B), the characteristic DOX fluorescence peaks were observed from PEI-rGO/DOX in 90% DMSO condition, in contrast to water condition where no DOX peaks were found. This result confirmed that DOX molecules would be bound to rGO surface tightly in water, although they would be dissociated from rGO surface in 90% DMSO by interference of the interactions between DOX and rGO. Based on the fluorescence result, the DOX loading efficiency of PEI-rGO/DOX was determined as 82.0%.

**Figure 4:**
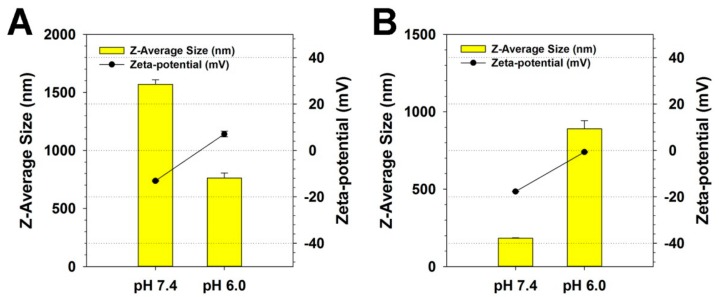
Z-average sizes and zeta-potential values of PKE(PEI-rGO/DOX) nanostructures at pH 7.4 and 6.0. (**A**) PK_5_E_7_(PEI-rGO/DOX) and (**B**) PK_5_E_9_(PEI-rGO/DOX). The PKE polymer coating ratio was set to a weight ratio of 30. Data are shown as the mean ± S.D (*n* = 3).

To examine the DOX loading effect to PKE(PEI-rGO)s, the average sizes and zeta-potential values of PKE(PEI-rGO/DOX)s were measured at pH 7.4 and 6.0 (Figure 4). The size of PK_5_E_7_(PEI-rGO/DOX) was 1570 nm at pH 7.4 and 762.0 nm at pH 6.0. Its zeta-potential value was −13.1 mV at pH 7.4 and increased to 7.1 mV at pH 6.0. PK_5_E_9_(PEI-rGO/DOX) was found to have 182.5 nm size at pH 7.4 and 889.9 nm at pH 6.0. Its zeta-potential was −17.7 mV at pH 7.4 and increased to −0.64 mV at pH 6.0. Considering the sizes and zeta-potential values of PKE(PEI-rGO) without DOX loading, a similar tendency responding to pH change was observed even after DOX loading. Since PK_5_E_7_(PEI-rGO/DOX) showed suitable charge-conversion behavior from pH 7.4 to 6.0, PK_5_E_7_(PEI-rGO/DOX) was focused on for further characterization in this work.

**Figure 5:**
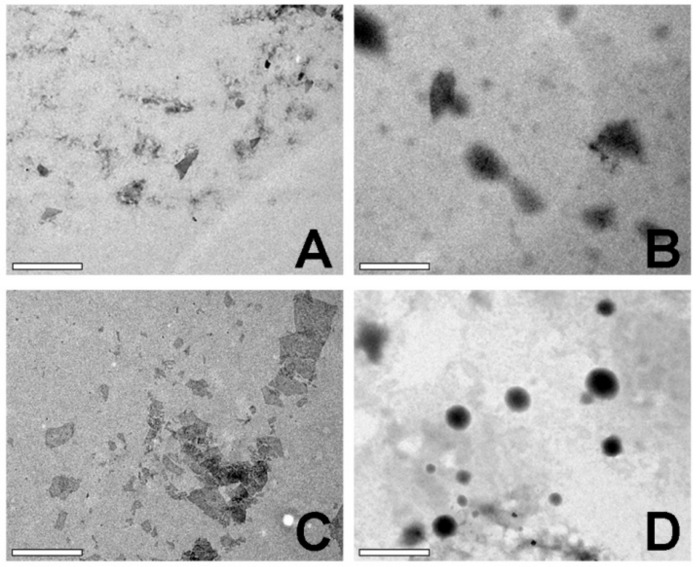
TEM images of (**A**) PEI-rGO, (**B**) PK_5_E_7_(PEI-rGO), (**C**) PEI-rGO/DOX, and (**D**) PK_5_E_7_(PEI-rGO/DOX). Scale bar = 0.5 μm.

The morphology of PEI-rGO nanostructures was observed by EF-TEM (Figure 5). In the case of PEI-rGO and PEI-rGO/DOX, plate-shaped nanostructures with sharp surfaces were observed (Figure 5A,C), showing the typical morphology of rGO. When the PK_5_E_7_ polymer was coated on PEI-rGO and PEI-rGO/DOX, spherical particles were observed (Figure 5B,D), showing a blurred edge characteristic of the polymer coating. This is a common phenomenon that occurs when carbon allotropes such as graphene or carbon nanotubes were coated or conjugated with polymers [30,33,36,37], which confirms that PK_5_E_7_ polymer was successfully coated on PEI-rGO and PEI-rGO/DOX. Additionally, the sizes of the nanostructures identified by TEM were approximately 300–400 nm, which are similar to the sizes measured by the zeta-sizer.

### 3.4. Serum Stability of PK_5_E_7_(PEI-rGO)

**Figure 6:**
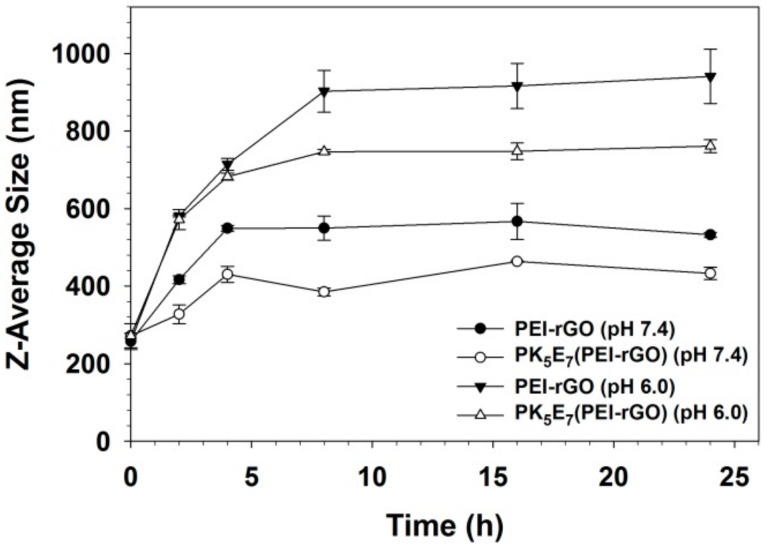
Z-average sizes of PEI-rGO and PK_5_E_7_(PEI-rGO) at pH 7.4 and pH 6.0 in serum condition (10% FBS). Data are shown as the mean ± S.D (*n* = 3).

In terms of drug or gene delivery systems, serum proteins in blood plasma are one of the most important obstacles to interrupt their therapeutic effects by the formation of large aggregates [38]. Therefore, the stability of PK_5_E_7_(PEI-rGO) nanostructure against serum proteins was examined by measuring its sizes under serum condition (10% FBS). As shown in Figure 6, the sizes of PEI-rGO and PK_5_E_7_(PEI-rGO) gradually were increased until 4–8 h of incubation, showing the interaction with serum proteins. However, in the case of PK_5_E_7_(PEI-rGO), the size of the nanostructures was much smaller than that of uncoated PEI-rGO nanostructure at both pH 7.4 and 6.0, meaning that PKE polymer coating could reduce the interaction with serum proteins. At pH 6.0, it was confirmed that the size of nanostructures was increased by increasing the electrostatic interaction of the positively-charged nanostructures with negatively-charged serum proteins, compared to the result at pH 7.4. The size of PK_5_E_7_(PEI-rGO) nanostructure was maintained as about 400 nm even after 24 h of incubation at pH 7.4, showing its high serum stability. In the absence of serum (Figure S2), PEI-rGO and PK_5_E_7_(PEI-rGO) showed similar sizes, although the sizes were increased in acidic condition probably due to the swelling by protonation of amines. Therefore, this result suggested that PEI-rGO nanostructure could be protected from non-specific interactions with anionic serum proteins at normal physiological pH via coating with charge-conversional PK_5_E_7_ polymer.

### 3.5. DOX Release Profile of PK_5_E_7_(PEI-rGO/DOX) 

**Figure 7:**
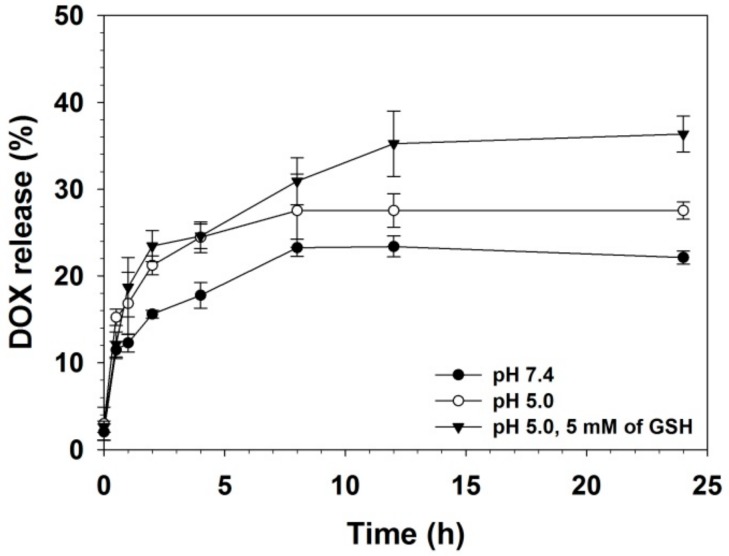
DOX release profile of PK_5_E_7_(PEI-rGO/DOX) in various condition (pH 7.4, pH 5.0, and pH 5.0 with 5 mM of GSH). Data are shown as the mean ± S.D (*n* = 3).

The drug delivery systems should release their cargo drug molecules to the desired location of cells under various conditions. The DOX release behavior of PK_5_E_7_(PEI-rGO/DOX) was investigated in three different conditions simulating the normal physiological condition (pH 7.4), the lysosomal condition after endocytosis (pH 5.0), and the lysosomal reducing condition after endocytosis (pH 5.0, 5 mM GSH) (Figure 7). DOX was released rapidly in all conditions until 2 h of incubation, meaning the initial burst release. After 2 h, DOX release at pH 7.4 was gradually increased from 15.6% and maintained as about 22% after 24 h, showing the lowest DOX release rate among all conditions. At pH 5.0, DOX release was increased, reaching about 27% after 24 h. However, DOX release at pH 5.0 in the 5 mM GSH condition was significantly higher (36.3%, 24 h) than DOX release at pH 7.4 and pH 5.0. This DOX release result indicated the pH- and GSH-responsive DOX release behavior of the PK_5_E_7_(PEI-rGO/DOX) nanostructure. In the acidic condition, DOX, PK_5_E_7_, and PEI-rGO were positively charged and their electrostatic repulsion would weaken the interaction between bound DOX and PEI-rGO. Additionally, hydrogen bonding between rGO and DOX would be strong in the neutral condition, which can suppress DOX release at pH 7.4 compared to pH 5.0 [39]. Thus, DOX molecules would be easily released from PK_5_E_7_(PEI-rGO/DOX) under the acidic condition.

DOX can be adsorbed on graphene materials via various mechanisms, such as π-π interactions, hydrogen bonds, and hydrophobic interactions [39,40,41]. It was reported that an intracellular reducing agent, GSH, can disturb hydrophobic and aromatic interactions between DOX and PEI-rGO, inducing DOX release in cytosol [33,42]. Therefore, it was expected that PK_5_E_7_(PEI-rGO/DOX) would release DOX efficiently after endocytosis, according to this result.

### 3.6. Anticancer Activity of PK_5_E_7_(PEI-rGO/DOX)

**Figure 8:**
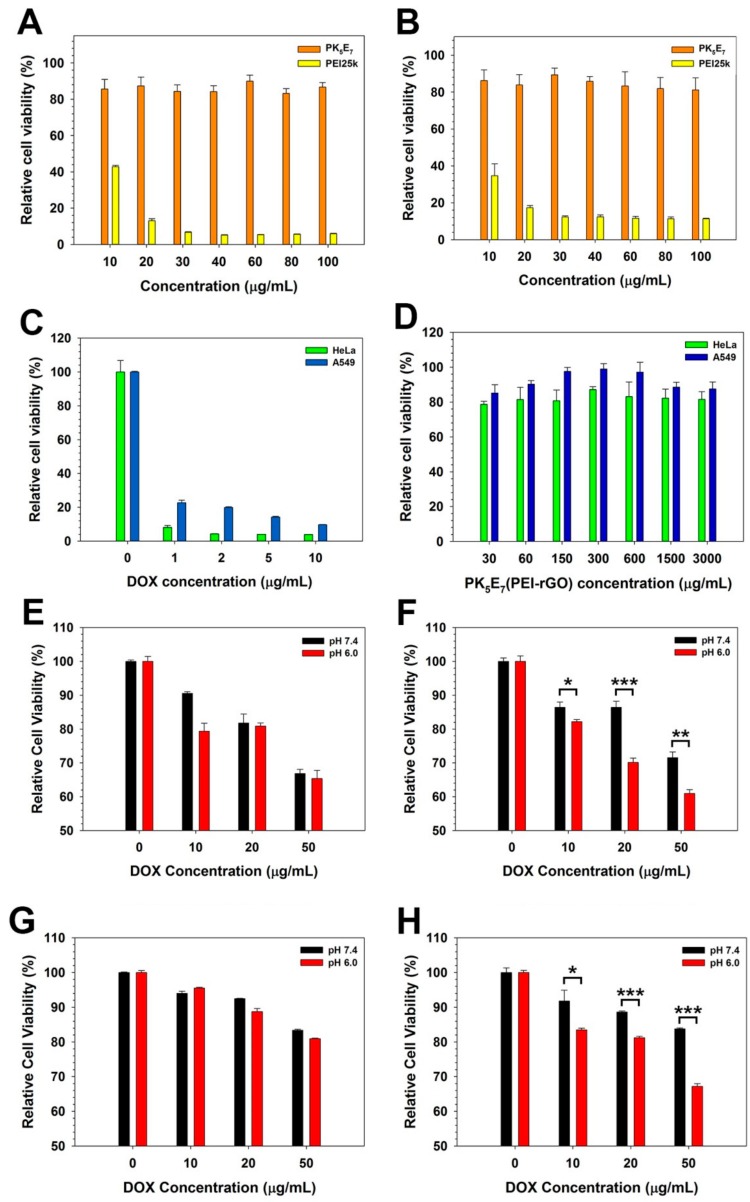
Cytotoxicity results of PK_5_E_7_ in HeLa cells (**A**) and A549 cells (**B**). PEI25k was used as a control. Anticancer activity of DOX only in HeLa and A549 cells (**C**). Cytotoxicity results of DOX-unloaded PK_5_E_7_(PEI-rGO) in HeLa and A549 cells (**D**). Anticancer activity of PK_5_E_7_(PEI-rGO/DOX) in HeLa (**E**,**F**) and A549 cells (**G**,**H**). The experiments were conducted in serum-free (**E**,**G**) and serum (10% FBS) condition (**F**,**H**). Data are shown as the mean ± S.D (*n* = 3) and statistically analyzed by *t*-test (* *p* < 0.05, ** *p* < 0.01, and *** *p* < 0.001).

Anticancer efficacy of PK_5_E_7_(PEI-rGO/DOX) was validated by MTT assay in HeLa and A549 cells (Figure 8). First, the low cytotoxicity of PK_5_E_7_ (Figure 8A,B) and DOX-unloaded PK_5_E_7_(PEI-rGO) (Figure 8D), and well-known cytotoxicity of DOX only (Figure 8C), were identified in HeLa and A549 cells. PEI25k controls showed concentration-dependent high cytotoxicity. PK_5_E_7_(PEI-rGO/DOX) showed DOX concentration-dependent decrease of cell viability in both serum-free and serum condition, regardless of cell types. Interestingly, much higher anticancer efficacy of PK_5_E_7_(PEI-rGO/DOX) was observed at pH 6.0 than pH 7.4 in serum condition, although no significant change of cell viability was found in serum-free condition at both pH 7.4 and 6.0 in HeLa cells (Figure 8D,E). The similar pH- and serum-responding anticancer activity behavior was also shown in A549 cells (Figure 8F,G). Lower anticancer activity of PK_5_E_7_(PEI-rGO/DOX) than DOX only was probably due to the slow release of DOX from the nanocarrier [43] and the association of released DOX molecules with negatively-charged PK_5_E_7_ polymers [44]. In addition, in the cased of DOX only, cells are exposed directly to DOX molecules with high concentration. However, without proper delivery systems possessing solvation and targeting ability, DOX concentration would be found to be very low in tumor tissue under in vivo conditions. It was also reported that free DOX could induce serious heart damage [45] and multi-drug resistance (MDR) of cancer cells could decrease the therapeutic effect by exporting internalized free DOX molecules [46] and that DOX-loaded nanocarriers could overcome MDR of cancer cells [47,48].

Contrary to pH 7.4, anionic serum proteins such as albumin would bind to PK_5_E_7_(PEI-rGO/DOX) at pH 6.0 due to its pH-responsive charge-conversion, according to the former results. Previously, it was reported that the human serum albumin (HAS)-coated lipoplexes could bind non-specifically to cell surface receptors, analogous to scavenger receptors, which mediate their endocytosis [49]. Another report also indicated the ability of albumin to promote membrane fusion under acidic conditions [50]. Therefore, it was suggested that albumin-decorated PK_5_E_7_(PEI-rGO/DOX) could bind to cell membranes non-specifically at pH 6.0 in serum condition, which would induce its facilitated endocytosis and DOX delivery to cancer cells, finally leading to the high anticancer activity. In addition, the increased DOX efficacy in acidic environment was probably due to the expedited DOX release from PK_5_E_7_(PEI-rGO) in acidic condition as shown in Figure 7. Therefore, it is expected that the PK_5_E_7_(PEI-rGO/DOX) nanostructure would show an enhanced cancer cell killing effect in the acidic tumor microenvironment condition via aid of decorated serum proteins, maintaining the intact status under normal physiological condition.

### 3.7. Cellular Uptake of PK_5_E_7_(PEI-rGO/DOX)

Flow cytometry analysis was performed to elucidate the pH and serum effect onto the cellular uptake of PK_5_E_7_(PEI-rGO/DOX) in HeLa and A549 cells (Figure S3). In HeLa cells (serum-free condition, Figure S3A), the cellular uptake efficiency of PK_5_E_7_(PEI-rGO/DOX) was 10.1% at pH 7.4 and it was decreased to 8.1% at pH 6.0. However, in the presence of serum (Figure S3C), the uptake efficiency was increased from 7.9% (pH 7.4) to 20.6% (pH 6.0). In A549 cells, similar cellular uptake behavior of PK_5_E_7_(PEI-rGO/DOX) was observed. The uptake efficiency was 27.8% at pH 7.4 and decreased to 8.1% at pH 6.0 in serum-free condition (Figure S3B). In serum condition (Figure S3D), it was increased from 18.6% (pH 7.4) to 31.8% (pH 6.0). These cellular uptake results were well correlated to the above anticancer activity of PK_5_E_7_(PEI-rGO/DOX).

In the serum-free condition, it was thought that relatively lower cellular uptake efficiency of PK_5_E_7_(PEI-rGO/DOX) at pH 6.0 than pH 7.4 would be induced by decelerated overall cellular activity [51] and by charge repulsion between positively-charged cell surface [52] and PK_5_E_7_(PEI-rGO/DOX) in acidic condition.

Interestingly, 1.7~2.6 times increased cellular uptake efficiency of PK_5_E_7_(PEI-rGO/DOX) at pH 6.0 in serum condition in comparison with the efficiency at pH 7.4, suggested that serum-protein-decorated PK_5_E_7_(PEI-rGO/DOX) by electrostatic interaction via charge-conversion would be internalized into cells more efficiently through non-specific interaction of albumin with cell membrane, overcoming the decelerated overall cellular activity and charge repulsion between positively-charged cell surface and PK_5_E_7_(PEI-rGO/DOX) in acidic condition.

**Figure 9:**
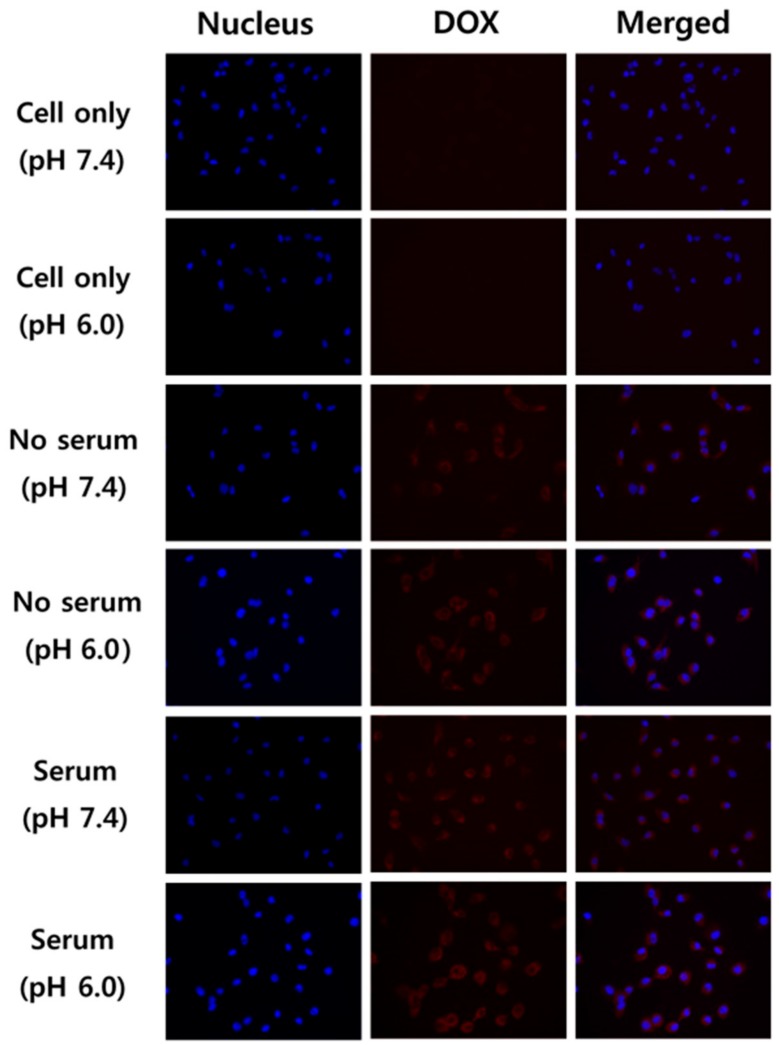
Fluorescence microscopy images of PK_5_E_7_(PEI-rGO/DOX) in A549 cells.

Cellular uptake behaviors of PK_5_E_7_(PEI-rGO/DOX) nanostructures were further observed by fluorescence microscopy (Figure 9). It was found that PEI-rGO/DOX could be internalized into cells even after PK_5_E_7_ polymer coating and that especially, in the acidic tumor microenvironment condition (serum, pH 6.0), PK_5_E_7_(PEI-rGO/DOX) displayed the highest cellular uptake activity, as shown in flow cytometry analysis result.

Overall, the scheme for the formation of PKE(PEI-rGO/DOX) nanostructures and their serum stability and decorated serum protein-mediated cellular uptake by PKE coating is presented in Figure S4.

## 4. Conclusions

In this work, DOX-loaded PEI-rGO was coated with pH-responsive charge-conversional polymer, PKE polymer, forming PKE(PEI-rGO/DOX) nanostructures and investigated for drug delivery systems. The pH-responsive charge-conversional property of PK_5_E_7_(PEI-rGO) was identified by zeta-potential value measurement. The sharp-edged sheet structure of PEI-rGO was transformed to spherical nanostructures with vague edges by PK_5_E_7_ coating. It was also found that PK_5_E_7_ coating could endow the stability against serum proteins to PK_5_E_7_(PEI-rGO) nanostructure. Loaded doxorubicin (DOX) in PK_5_E_7_(PEI-rGO) could be released rapidly in lysosomal condition (pH 5.0, 5 mM glutathione). Furthermore, DOX-loaded PK_5_E_7_(PEI-rGO) showed enhanced anticancer activity in cancer cells in tumor microenvironment-mimicking condition, which would be facilitated by improved cellular uptake via non-specific cellular interaction with decorated serum proteins. Therefore, pH-responsive charge-conversional PKE polymer-coating strategy of cationic nanostructures is thought to possess a potential for tumor microenvironment-targeted drug delivery systems.

## Supplementary Materials

The following are available online at https://www.mdpi.com/2079-4991/9/9/1289/s1, **Figure S1.** Size distribution histograms of PK_5_E_7_(PEI-rGO) nanostructures, **Figure S2.** Z-average sizes of PEI-rGO and PK_5_E_7_(PEI-rGO) at pH 7.4 and pH 6.0 in PBS buffer, **Figure S3.** Cellular uptake analysis of PK_5_E_7_(PEI-rGO/DOX) by flow cytometry in HeLa and A549 cells, **Figure S4.** The scheme for the formation of PKE(PEI-rGO/DOX) nanostructures and their serum stability and decorated serum protein-mediated cellular uptake by PKE coating.
