# Supplementary material for: Effect of pH-Responsive Charge-Conversional Polymer Coating to Cationic Reduced Graphene Oxide Nanostructures for Tumor Microenvironment-Targeted Drug Delivery Systems
Source: Nanomaterials (Basel). 2019 Sep 9;9(9):1289. doi: 10.3390/nano9091289 (PMC6781034; doi:10.3390/nano9091289)
Supplement: Supplementary file 1 [file nanomaterials-09-01289-s001.pdf]

nanomaterials

MDPI

Supplementary materials

Effect of pH-Responsive Charge-Conversional Polymer Coating to Cationic Reduced Graphene Oxide Nanostructures for Tumor Microenvironment-Targeted Drug Delivery Systems

Kitae Ryu [1], Jaehong Park [1], Tae-il Kim[1,2,*]

[1] Department of Biosystems & Biomaterials Science and Engineering, College of Agriculture and Life Sciences, Seoul National University, 1 Gwanak-ro, Gwanak-gu, Seoul 08826, Korea

[2] Research Institute of Agriculture and Life Sciences, Seoul National University, 1 Gwanak-ro, Gwanak-gu, Seoul 08826, Korea

* Correspondence to: seal1004@snu.ac.kr

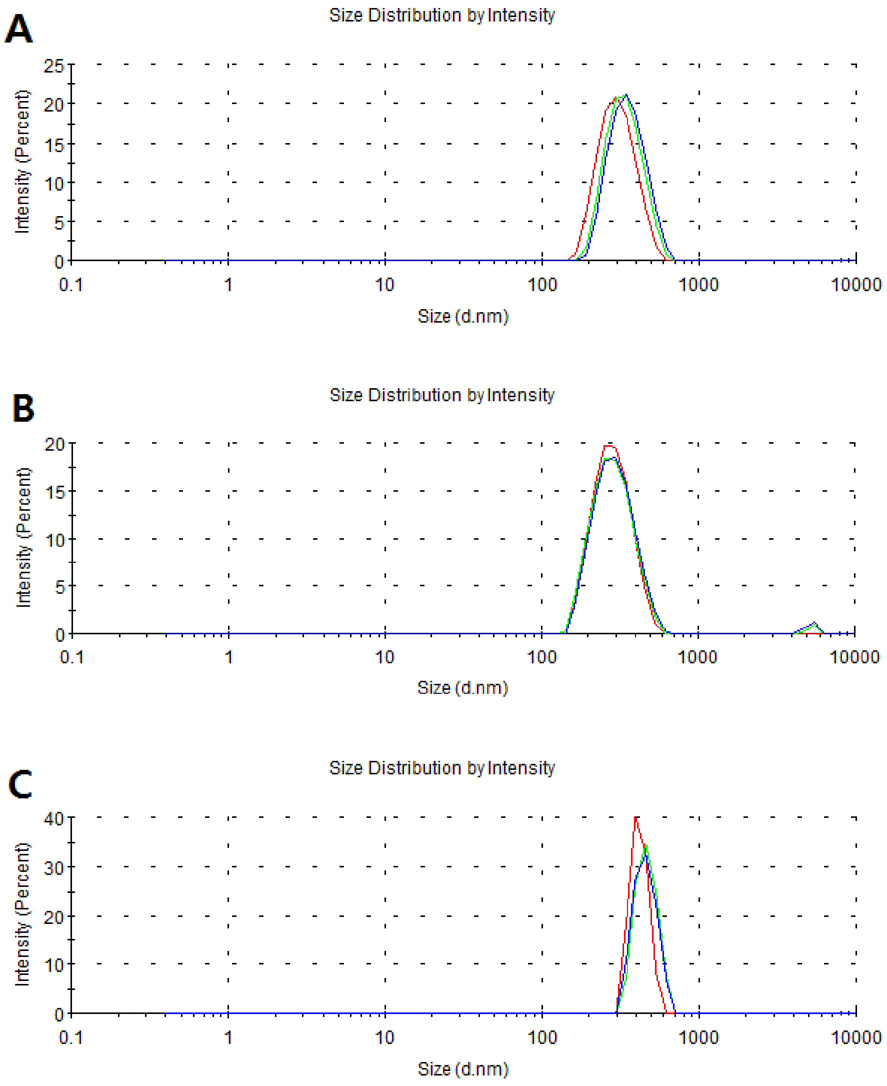

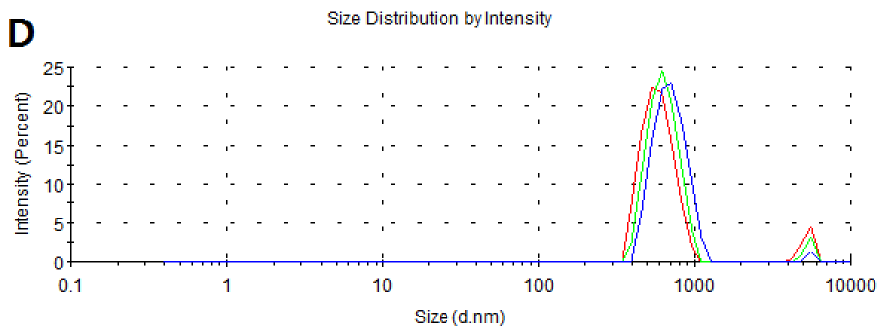

Figure S1. Size distribution histograms of PK_5E_7(PEI-rGO) nanostructures. (**A**) Weight ratio (PK_5E_7/PEI-rGO) = 10, PDI = 0.190 ± 0.053, (**B**) weight ratio = 20, PDI = 0.180 ± 0.021, (**C**) weight ratio = 30, PDI = 0.409 ± 0.088, and (**D**) weight ratio = 50, PDI = 0.349 ± 0.055. Size measurements were performed in triplicate.

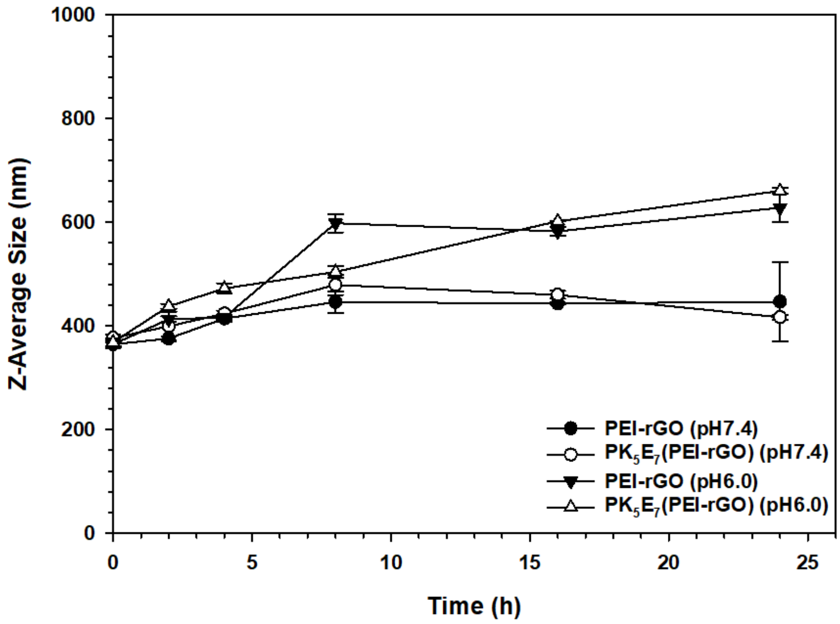

Figure S2. Z-average sizes of PEI-rGO and PK5E7(PEI-rGO) at pH 7.4 and pH 6.0 in PBS buffer. Data were shown as mean ± S.D (n=3).

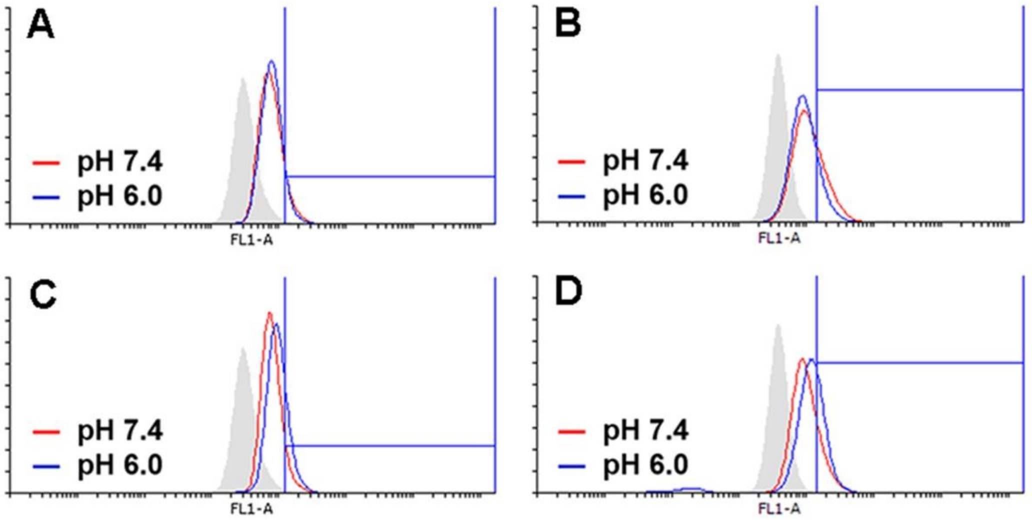

Figure S3. Cellular uptake analysis of PK5E7(PEI-rGO/DOX) by flow cytometry in HeLa (**A**, **C**) and A549 cells (**B**, **D**). The experiments were conducted in serum-free (A, B) and serum (10% FBS) condition (C, D). Gray peaks present cell only results.

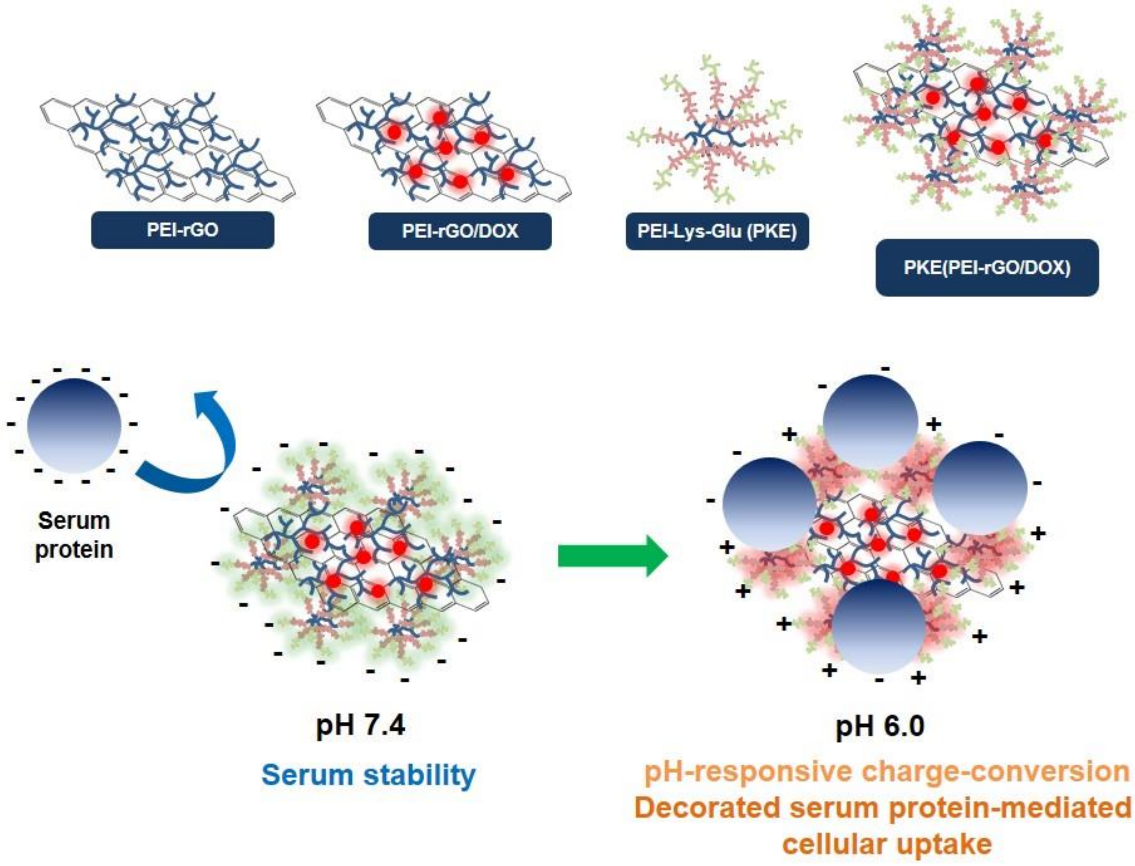

Figure S4. The scheme for the formation of PKE(PEI-rGO/DOX) nanostructures and their serum stability and decorated serum protein-mediated cellular uptake by PKE coating. It suggests that the pH-responsive charge-conversional PKE polymer coating strategy of cationic rGO nanostructures possesses a potential for acidic tumor microenvironment-targeted drug delivery systems.
